# Antigen and Transforming Growth Factor Beta Receptors Contribute to Long Term Functional and Phenotypic Heterogeneity of Memory CD8 T Cells

**DOI:** 10.3389/fimmu.2013.00227

**Published:** 2013-08-12

**Authors:** Yinghong Hu, Linda Cauley

**Affiliations:** ^1^University of Connecticut Health Center, Farmington, CT, USA

**Keywords:** transforming growth factor beta, CD8 T cells, homing receptors, prolonged antigen presentation, tissue-resident memory cells, migration

## Abstract

Pathogen-specific CD8 T cells provide a mechanism for selectively eliminating host cells that are harboring intracellular pathogens. The pathogens are killed when lytic molecules are injected into the cytoplasm of the infected cells and begin an apoptotic cascade. Activated CD8 T cells also release large quantities of pro-inflammatory cytokines that stimulate other immune cells in the local vicinity. As the alveoli are extraordinarily sensitive to cytokine induced damage, multiple layers of immune regulation limit the activities of immune cells that enter the lungs. These mechanisms include receptor-mediated signaling pathways in CD8 T cells that respond to peptide antigens and transforming growth factor β. Both pathways influence the functional and phenotypic properties of long-lived CD8 T cells populations in peripheral and lymphoid tissues.

Adaptive immune responses to new pathogens begin after naïve T cells encounter mature DC with their cognate antigen in the secondary lymphoid organs. Extensive phenotypic and functional changes occur as the T cells progress along a complex differentiation pathway (Figure 1). Some of the earliest changes include the loss of homing receptors that are required to enter the encapsulated lymph nodes, which are replaced by other molecules that guide activated T cells into infected tissues. Many functional properties are also modulated during exposure to antigen or environmental stimuli, leading to the acquisition of new effector functions and altered capacity for long term survival (1). The enduring characteristics of the surviving memory T cells sometimes reflect partial progression along a chosen differentiation pathway after weak antigen stimulation, insufficient costimulation, or limited inflammation (2).

**Figure 1:**
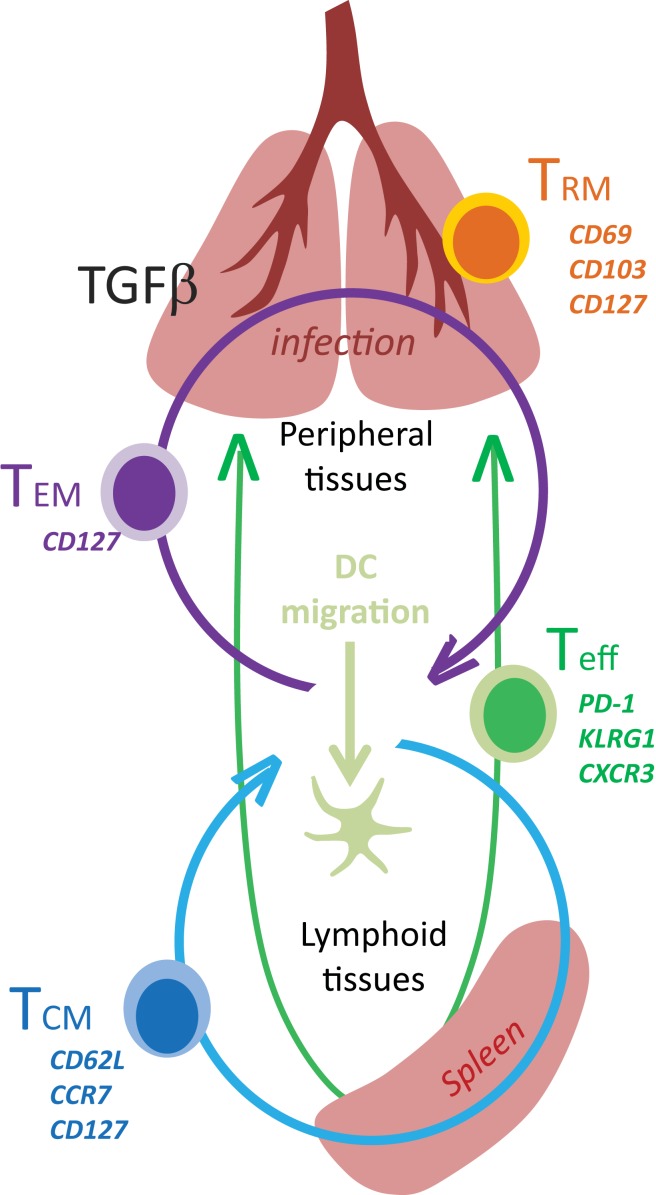
**Tissue distribution of memory CD8 T cells**.

## The Phenotypic Characteristics of Naïve CD8 T Cells

The secondary lymphoid organs serve as centralized sites of immune activation and accommodate large numbers migratory DC which carry microbial products from infected tissues (3, 4). Rare antigen-specific T cells provide comprehensive immune surveillance by moving sequentially between different lymphoid tissues until they encounter antigen presenting cells (APCs) with their cognate antigen. Some circulating lymphocytes (including naïve CD8 T cells) enter encapsulated lymph nodes by squeezing between cuboidal endothelial cells that line wide vessels known as high endothelial venules (HEV) (5). The migrating cells express L-selectin (CD62L) which interacts with peripheral lymph node addressins (pNAD) causing the T cells to begin rolling over the surface of the endothelial cells (6, 7). The rolling T cells constitutively express CC chemokine-receptor 7 (CCR7) and respond to chemokines ccl19 and ccl21 (8, 9) which promote a conformational change in the structure of an integrin known as leukocyte adhesion molecule-1 (LFA-1) (10–12). Tight interactions between activated LFA-1 and Intercellular Adhesion Molecule-1 (i.e., ICAM-1) are essential for diapedesis (13, 14). After crossing the endothelial layer, naive T cells use conduits of reticular cells which are coated with ccl19 and ccl21 to search for DC with their cognate antigen (9, 15, 16).

## Antigen Stimulation Leads to Extensive Phenotypic and Functional Changes

All nucleated cells can assemble MHCI molecules using peptides from self-derived proteins however the mechanisms that are used to produce antigenic peptides from foreign proteins are not identical for all cell types (17, 18). Infected cells produce defective ribosomal products which are directed to the proteasomes for degradation and are pumped from the cytosol into the endoplasmic reticulum by the Transporter for Antigen Presentation (TAP) where the complete peptide/MHCI complexes are assembled. Other APCs (i.e., some DCs and macrophages) acquire foreign proteins from cells in the surrounding tissues and produce immunogenic peptides without infection which are used for cross-presentation to CD8 T cells (19, 20). The preferential use of a specific peptide processing pathway can influence the specificity of the CD8 T cell response and alter the pattern of epitope dominance during some infections (21).

At least two subsets of migratory DC carry microbial products into encapsulated lymph nodes and other lymphoid tissues (22). Other DCs are permanent residents of the lymph nodes and acquire antigens from neighboring cells (20, 23). These DC express a variety of coreceptors that exert positive or negative effects on T cells during antigen stimulation, but play little or no role in the immune response unless the TCR is engaged. The coreceptors that augment Teff functions, including cytokine production and lytic activity, are known as costimulatory molecules while inhibitory receptors suppress functional activities and cell cycle progression (24). Some important costimulatory signals are delivered through CD28 which interacts with CD80 and CD86 during the formation of the immunological synapse (25, 26). Clonal diversity can be increased by costimulation through CD27, which promotes cell survival during responses to low affinity antigens (27, 28). Other coreceptors are induced by TCR derived signals and modulate the properties of responding T cells as the infection progresses (29). Costimulation through 4-1BB, OX40, or CD27 leads to increased expression of anti-apoptotic molecules such as BCL-2 and BCL-XL and prolonged T cell survival (30), while CD30 has pleiotropic effects on T cell activation, apoptosis, and effector function.

Antigen stimulation causes many external changes as naïve CD8 T cells become Teff cells. Some permanent changes include increased CD44 and LFA-1 (CD11a) expression, which are required for activated T cells to enter peripheral tissues (11, 31–33). Other surface molecules are reversibly induced during antigen stimulation including chemokine receptors which control the distribution of antigen-specific CD8 T cells in inflamed tissues, such as CXCR3 (34, 35). Some activated T cells leave the blood vessels using chemokine-dependent mechanisms, however a recent study has shown that cognate antigen can induce transendothelial migration in vascularized transplants by a mechanism that is independent of Gαi-signaling (36). Other surface molecules are down regulated during antigen stimulation including CCR7 and CD62L which can be cleaved from the cell surface by metalloproteases (37). Foxo-1 plays a role in the transcriptional control of CCR7 and CD62L expression in T cells (38).

The functional characteristics of CD8 T cell populations are modified by cell-fate decisions during memory development. Some experiments indicate that asymmetric cell division determines the ratios of Teff cells and memory cells (39). Others suggest that the strength of the TcR signal determines whether CD8 T cells undergo symmetric or asymmetric cell division and thus controls the phenotype of the daughter cells (40, 41). This idea was not supported by transfer studies with individual OTI cells which express a high-affinity TcR and produced heterogeneous progeny after infection (41–43). Some experiments suggest that naïve CD8 T cells become T_CM_ precursors (Tcmp), before becoming T_EM_ precursors (Temp) and finally Teff cells (43). This linear differentiation model is supported by the finding that Tcmp proliferate slower than the Temp or Teff cells (43). The model can be reconciled with data which show that recurrent antigen stimulation or inflammation increases the percentages of short-lived Teff cells within the population, while virus-specific CD8 T cells that are activated later in the response may receive less stimulation and preferentially differentiate into the Tcm phenotype (44). The disparate fates of progeny cells from individual parent T cells underscore the importance of extrinsic signals during memory differentiation, which can come from a variety of sources including the APCs, costimulatory molecules, or cytokines.

## Cytokines Contribute to the Heterogeneity of Activated T Cell Populations

Recent studies have shown that IL-1 is not only critical for the activation of DCs (45), but also significantly increases clonal expansion and augments the effector functions of virus-specific CTL (46). During the expansion phase of the infection, autocrine IL-2 production is essential for Teff cell differentiation and survival. The IL-2 derived signals promote sustained Blimp-1 expression and repress Bcl-6 (47) which sustains mTOR activity and glycolysis via the PI3K-Akt pathway (48). Some Teff cells maintain CD25 expression (i.e., the high-affinity IL-2 receptor) and undergo extensive proliferation before becoming terminally differentiated Teff cells, while other cells lose CD25 and maintain the capacity to become memory cells (49). Large numbers of Teff cells that express the Killer cell lectin-like receptor G1 (KLRG1) but not CD127, die during contraction of the CTL response and are known as short-lived effector cells (SLECs) (31). Other cells which lack KLRG1 and re-express CD127 before the contraction begins, are known as memory precursor effector cells (MPECs) because they are more resistant to apoptosis. KLRG1 is a useful phenotypic marker however expression is not required for Teff differentiation or development of robust effector functions (50). Two inhibitor of DNA binding proteins (Id2 and Id3) influence memory CD8 T cell development before the phenotypic markers of MPECs and SLECs change. Both proteins inhibit E-protein transcription factors but they promote CD8 T cell survival by different mechanisms (51–54). Specifically Id2 supports the survival of Teff cells by inducing anti-apoptotic molecules such as Bcl-2, while reducing the expression of pro-apoptotic molecules such as Bim (51, 52). In contrast Id3 prolongs the survival of memory cells by regulating key genes that are essential for genomic stability (53, 54).

The milieu of pro-inflammatory cytokines that are produced upon innate immune recognition of pathogen-associated molecular patterns (PAMPs) can also influence the functional properties of developing CD8+ Teff cells. For example, IL-12 or type I interferon (IFN-I) can lead to STAT4 phosphorylation and T-bet expression which promotes terminal differentiation of SLECs (55). In addition, IL-12 activates the PI3K-Akt-mTOR pathway which drives rapid proliferation of Teff cells and promotes degradation of Foxo-1, which in turn leads to the down regulation of Eomesodermin (Eomes) and loss of CD127, CD62L, and CCR7 (56). The T-bet and Eomes transcription factors also regulate CD8 T cell effector functions, as shown by high IL-17 expression and excessive leukocyte infiltration when these molecules are not expressed (57). As the levels of pro-inflammatory cytokines decline, IL-10 and IL-21 activate STAT3 to promote memory development by inducing Bcl-6, Eomes, and suppressor of cytokine signaling 3 (SOCS3) (58). SOCS3 expression may be essential for preserving memory potential by dampening the IL-12 response and shifting their metabolic state back to oxidative phosphorylation as the activated CD8 T cells become quiescent.

Most newly activated Teff cells are capable of immediate lytic activity and cytokine expression, but have a very short life span. Members of the common γ-chain cytokine family play a complex role in CD8 T cell survival and elicit responses that can be modulated through changing receptor expression. The loss of CD127 expression on naive CD8 T cells is partly controlled by the Foxo-1 transcription factor, which can be inactivated via the PI3K-Akt-mTOR signaling pathway (50). Some activated T cells re-express CD127 before the peak of the CTL response and have an enhanced capacity to become long-lived memory cells (59). Re-expression of CD127 is controlled by the transcription factor GA binding protein α (GABPα) which is responsible for hyperacetylation of the promoter, while growth factor independence 1 (Gfi-1) is an antagonist that suppresses CD127 expression on late Teff cells by recruiting histone deacetylase 1 (60). The upstream signaling molecules that regulate GABPα and Gfi-1 expression have not been clearly defined.

Multiple mechanisms contribute to the contraction of Teff response, including the withdrawal of essential growth factors such as IL-2 (48) and perforin or TGFβ induced apoptosis (61, 62). Only small percentages of Teff cells have the capacity to survive through the contraction and become long-lived memory cells. Cell survival is determined by a delicate balance between of pro-survival molecules such as Bcl-2 or Mcl-1, with pro-apoptotic molecules such as Bim or Noxa, which can be regulated by external signals in the tissues (63–65). A recent study has shown that some pro-apoptotic signals are induced by TGFβ, but can be antagonized by the pro-survival properties of IL-7 and IL-15 (62). Forced CD127 expression does not prevent contraction of the Teff population (66) which indicates that terminally differentiated SLECs have an intrinsic defect in their response to IL-7 signaling, as suggested by high expression of the cell cycle inhibitor p27Kip (43). Consequently IL-7 in combination with IL-15 promotes the survival of MPECs, while SLECs are critically dependent on the stimulation through the IL-2/IL-15 receptor (67).

## Chronic Antigen Stimulation Promotes Phenotypic and Functional Heterogeneity in CD8 T Cells

CD69 and PD-1 are surface proteins that are transiently induced on activated CD8 T cells soon after TcR stimulation (68, 69). The function of CD69 is not known, but some studies suggest that interactions between CD69 and the sphingosine-1-phosphate receptor-1 (S1P1) facilitate efficient migration of activated CD8 T cells into the bloodstream (70). CD8 T cells transiently express CD69 in infected tissues when IFN-I is present, however expression levels quickly decline when the cytokine is removed (71). PD-1 is also expressed on activated T cells during antigen stimulation but expression cannot be induced by IFN-I. PD-1 disappears when the antigen is removed and is thus a reliable indicator of persisting peptide/MHC complexes.

When CD8 T cells are exposed to a continuous supply of antigen during chronic infections or inside tumors they adopt an altered phenotype which is characterized by high level PD-1 expression together with other inhibitory coreceptors such as TIM3, CTLA4, BTLA, CD160, LAG3, and 2B4 (72). The responses of CD8 T cells that express one or more of these inhibitory receptors are attenuated as shown by reduced proliferative capacity and tempered effector functions, which led to the term “exhausted” T cells (73). Interactions with PD-1 ligands can impair CD8 T cell functions through multiple mechanisms, including reduced mobility (74). The symptoms of exhausted CD8 T cells were reversed in some studies, using combinations of antibodies to block interactions with PD-1 and other inhibitors such as TIM3, CTLA4, and/or LAG3 (72). Large numbers of exhausted CD8 T cells are often accompanied by depleted populations of memory CD8 T cells, which suggests that they may be the product of chronically stimulated Teff cells. Evidence that specific APCs play a role in the development of exhausted CTL has not been reported but since the cells do not express KLRG1 suboptimal differentiation may play a role (75). Indeed, network analysis recently revealed fewer transcriptional modules of quiescence in exhausted CD8 T cells, as compare to functional memory cells (76). In contrast to memory CD8 T cells, exhausted CTL are maintained in an antigen-dependent manner and gradually disappear when they are transferred to infection-free mice (77). Most functional studies have focused on the properties of exhausted CD8+ T cells however there is evidence that CD4+ T cells can exhibit symptoms of exhaustion in some situations (26).

## The Phenotypic Properties of Long Live Memory CD8 T Cells

Two major subsets circulating memory CD8 T cells survive the contraction of the Teff response and can be distinguished using reciprocal CD62L and CCR7 expression (78). Central memory (T_CM_) CD8 T cells are CD62L+CCR7+ cells that can access secondary lymphoid organs via HEV and have a similar tissue distribution as naive CD8 T cells (Table 1). Since effector memory CD8 T cells (T_EM_) lack CD62L and CCR7 expression they cannot access encapsulated lymph nodes under steady state conditions, however some activated CTL can access inflamed lymph nodes during infection by a mechanism that requires CXCR3, but not CD62L (79). Recent studies have shown that a third major subset of memory CD8 T cells resides in selected peripheral tissues after local infections and does not return to circulation after inflammation subsides (80). The highest concentrations of these tissue-resident memory (T_RM_) CD8 T cells are typically found in tissues with an epithelial layer, during the recovery from a recent infection (81–84). Some studies indicate that recent exposure to cognate antigens plays a role in the long term retention of CD8+ T_RM_ cells in tissues such as the lungs and CNS, where some KLRG1-negative CTL express CD103 (αeβ7 integrin) when activated TGFβ is present (81, 85, 86). Other studies indicate that sustained antigen exposure is dispensable for maintenance of T_RM_ cell in the gastrointestinal tract (87). The influence of pathogen-derived peptides on lymphocyte migration is controversial since several viruses which were previously thought to induce “acute infections” leave residual peptides that persist *in vivo* for weeks or months after inoculation (88–90). Additional peptides may persist longer but are below the level of detection. Although the reasons for the heterogeneous characteristics of pathogen-specific memory CD8 T cells *in vivo* have not been clearly defined, the duration of the infection and the pathogen’s capacity to elicit specific cytokines can have a dramatic influence on the enduring characteristics of the response.

**Table 1:** **Phenotypic heterogeneity of CD8 T cell subsets**.

	CD44	CD62L	KLRG1	CCR7	CD69	PD-1	CD103	CD25	CD127	CD122
Naive	±	∙		∙			±		∙	
Teff	∙		∙		∙	∙		∙		∙
T_CM_	∙	∙		∙					∙	∙
T_EM_	∙								∙	∙
T_RM_	∙				∙	±	∙		∙	
Exhausted	∙				∙	∙				∙

*∙ indicates marker expressed at high levels*.

Stable CD69 and CD103 expression are hallmarks of T_RM_ cells that can be found in the skin, gastrointestinal tract, and lungs (80, 91). Some studies suggest that epithelial cells provide signals for sustained CD69 expression, which does not require chronic antigen stimulation (87, 92). Whether CD69 influences the distribution of T_RM_ cells in peripheral tissues such as the lungs (81) through interactions with the sphingosine-1-phosphate (S1P) receptor-1 remains to be determined (93). Others found that an ongoing response to antigen stimulation was required for T_RM_ cells to maintain stable CD103 expression in the lungs (81) and CNS (85). Additional evidence of a prolonged response to antigen stimulation by T_RM_ cells in the lungs includes low level expression of PD-1 (94) and interferon-induced transmembrane protein 3 (IFITM3) (95), while CD103 expression declined when antigen-specific antibodies were used to block TcR interactions with peptide/MHC complexes (81). T_RM_ cells in the brain also expressed CD103 only after intracerebral inoculation with Vesicular stomatitis virus (VSV) (81, 95).

## Transforming Growth Factor-β and Heterogeneity of CD8 T Cells in Mucosal Tissues

Transforming growth factor-β1 (TGFβ1) is pleiotropic cytokine that plays a central role in immune homeostasis. The regulatory properties of TGFβ include potent anti-proliferative and pro-apoptotic effects on virus-specific CD8 T cells, which contribute to the contraction of the Teff response during some infections (62). Teff cells are resistant to apoptosis during clonal expansion, but become highly vulnerable to deletion after KLRG1 is upregulated (62). Very few KLRG1+ CD8 T cells survive in the lungs during infections with some strains of influenza and other respiratory viruses that make enzymes which can activate TGF-β (96–99). Paradoxically, exposure to activated TGFβ leads to αEβ (7) integrin (CD103) expression on long-lived CD8+ T_RM_ cells, which often reside near epithelial cells that express E-cadherin (81, 100).

The reasons why individual subsets of CD8 T cells respond to TGFβ in different ways is not known, but multiple different signaling pathways may play a role (79, 80). The apoptotic effects of TGFβ on Teff cells can be overcome by IL-2 and partially inhibited by IL-7, but IL-15 has no protective value (62). This reason why TGFβ exerts its pro-apoptotic role after the peak of the Teff response may be due to the presence of IL-2R (CD25) at earlier time points. This may also explain why SLECs are particularly sensitive to TGFβ-induced apoptosis, as this subset lacks CD127 and depends on IL-15 for survival. The ability of γc cytokines to antagonize the apoptotic effects of TGFβ signaling may be determined by their ability to activate the PI3K pathway, which interacts with TGFβ-induced Smad proteins in a complex manner. Activated Akt can directly associate with Smad3 and inhibit phosphorylation by TGFβRI, which prevents translocation into nucleus. Also, p15Ink4b and p21Cip1 are inhibitors of cyclin-dependent kinases, which can be induced by TGFβ and are required the formation of a transcription complex that is composed of Smad3, Smad4, and the Foxo transcription factors. The PI3K-Akt pathway can induce phosphorylation and degradation of Foxo proteins, and thus antagonize the inhibitory effect of TGFβ during cell cycle progression. On the other hand, TGFβ signaling can dampen the PI3K pathway through the induction of lipid phosphatase SHIP. TGFβ signaling can also dephosphorylate S6K downstream of PI3K-Akt-mTOR pathway via the induction of protein phosphatase 2A (PP2A) (83).

The signaling pathways that are activated during TGFβ regulation are more clearly defined for CD4 than CD8 T cells. Studies have shown that TGFβ induces Sma and Mad-related (SMAD) transcription factors to repress Id3 and enhance binding of E2A in CD4 T cells, which is crucial for the induction of the forkhead box p3 (Foxp3) gene (101) and inhibits the development of Th1 cells (102). Other signaling pathways include the MAP kinase (MAPK), Rho-like GTPase, and phosphatidylinositol-3-kinase (PI3K) pathways (103). A master transcription factor RORγt can be induced in CD4 T cells from mice that lack either Smad4, or Smad2 and Smad3 expression (102). TGFβ also promotes Th17 development by suppressing Eomes via the c-Jun N-terminal kinase (JNK)-c-Jun signaling pathway (104). Since some pathogens elicit robust TGFβ responses it is likely that these signaling pathways have a dramatic influence on the activities of pathogen-specific CD8 T cells during infection, which play a critical role immunity in mucosal tissues.

## Summary

Together the current data show that the cytokine milieu and prolonged presence of foreign antigens are responsible for extensive heterogeneity in long-lived CD8 T cell populations. This heterogeneity is reflected by a broad tissue distribution and diverse functional properties which are absolutely essential to combat an enormous variety of different pathogens.

## Conflict of Interest Statement

The authors declare that the research was conducted in the absence of any commercial or financial relationships that could be construed as a potential conflict of interest.
